# Caring for People at the End of Life: Iranian Oncology Nurses' Experiences

**DOI:** 10.4103/0973-1075.58461

**Published:** 2009

**Authors:** Sedigheh Iranmanesh, Abbas Abbaszadeh, Helen Dargahi, Mohammad Ali Cheraghi

**Affiliations:** Faculty of Nursing and Midwifery, Kerman University of Medical Sciences, Kerman, Iran; 1 Valiasr Hospital, Tehran University of Medical Science, Tehran, Iran; 2 Faculty of Nursing and Midwifery, Hamedan University of Medical Sciences

**Keywords:** Caring for dying people, Lived experience, Iran, Oncology nurses, Palliative care

## Abstract

**Aim:**

To explore the meaning of Iranian oncology nurses' experiences of caring for people at the end of life.

**Materials and Methods:**

A phenomenological hermeneutic approach was applied. Fifteen nurses working in oncology units were interviewed in 2007 regarding their experiences of caring for people at the end of life.

**Results:**

Participants experienced caring for people at the end of life as sharing space and time to be lost within an organizational context. This main theme was divided into three subthemes including being attentive to the dying persons and their families, being cared for by the dying persons and their families, and being faced with barriers.

**Conclusion:**

The study suggests that the nurses' success in caring for people at the end of life is reliant on their interpersonal caring relationship. Facilitating such relationship requires the establishment of palliative care unit, incorporation of palliative care into undergraduate nursing studies, and cultural preparation through public education.

## INTRODUCTION

The burden of cancer in the worldwide context continues to grow, with an increasing number of new cases and deaths each year.[1] Oncology nurses deal with death, even while helping patients to live each day to the fullest.[2] In many ways, death presents the greatest challenge to care. Death causes an existential crisis for many people, especially those who are facing a life-threatening disease. According to Sand and Strang,[3] the inevitability of death triggers an existential crisis involving patients' and families' emotions and perceptions. The loss of future and the approach of death make it difficult for dying persons to establish the meaning of life and being. These issues force terminally ill persons to experience existential pain as a loss of identity and meaninglessness of life.[4] Such an existential crisis requires a holistic approach to be able to meet all patients' concerns. Furthermore, cancer is not a disease of an individual but it impacts the whole family system; family members may assume new roles and responsibilities.[5] The family members who have a supportive role often become caregivers and exhaust themselves, and therefore they need professional services and support.

Death also presents the greatest challenge to care. The perception of caring for dying persons involves nurses having professional attitudes and skills in order to provide good care. This includes emotional and practical support.[6] According to Keegan *et al.*,[7] the dying persons' relatives consider all aspects of care to be important toward the end of life. However, they pay special attention to the attitudes with which care is given and how it preserves the patient's dignity. Studies exploring the experience of being a family member of a dying person[8,9] have revealed the family members' grieve with feelings of sadness and helplessness. They need active informational and emotional support from nurses.

Some sporadic published nursing studies have actually explored the phenomenon of caring for dying people among oncology nurses. According to the available literature, one of the first phenomenological studies was conducted by Rittman *et al.*,[2] by analyzing the oncology nurses' stories of caring for dying patients. Their findings were categorized into four themes: “Knowing the patient”, “preserving hope”, “easing the struggle,” and “providing for privacy”. Furthermore, to shed lights on the oncology nurses' perceptions of caring for dying patients, Pavlish and Ceronsky[10] recently carried out a descriptive study with narrative data analysis. Three descriptors of the healthcare context in which palliative care occurs were identified: Limited time for addressing complex palliative care issues, health care's emphasis on prolonging life, and the challenge of coordinating care across disciplines.

The studies referred earlier somewhat tried to explore the understanding of the phenomenon of caring for dying people by using different methods of analysis for the nurses' experiences in oncology settings. It is, therefore, the aim of this study to add to this body of knowledge by referring to the experiences of a group of oncology nurses in Iran.

Iran, the home of one of the most ancient world civilizations, is part of a Middle East culture. The country with a population of 68 million has over 120,000 nurses, out of which 70,000 are providing nursing care in general and specialist hospitals. Consequently, the country is faced with a nursing shortage, which is forcing the nurses to work beyond their regular shift.[11] The nursing program includes general education courses, professional foundation courses with a biological content, and nursing courses with a biomedical nursing content.[12] Despite this, there is a belief among nurses that holistic care is the essence of nursing and is seen as a motivating factor to continue their work.[13] Given that cancer accounts for a major cause of death, oncology care has been developing with time. The curriculum content in the BS, MS, and PhD degrees includes 0.5 credits equal to 8 h about oncology care. End of life care is still a new topic in Iranian health care system; hence nurses are not educated in that either clinically or academically, even those who are involved with people at the end of life. This lack of education and experience, as well as some cultural and professional limitations, contributed to the attitudes of Iranian nurses toward caring for people at the end of life.[14]

## MATERIALS AND METHODS

Using a phenomenological approach, phenomena are studied from the viewpoint of the individual. The methodology seeks to focus on a person's experience. It is based on that person's previous understanding and knowledge, which are embedded in culture and history. Husserl[15] described phenomenology as a turn to the things themselves. It was described as a view of the world based on experience. The overall aim of life-world research, according to Dahlberg *et al.*,[16] is to describe and elucidate the lived world in a way that expands our understanding of human being and human experience. In this sense, phenomenology and hermeneutics are the prerequisites for each other.[17] Phenomenological hermeneutics helps us to develop the critical understanding of a studied discourse. In other words, it helps us to obtain knowledge of the essential meaning of a lived experience.[18]

### Participants

Purposeful sampling was used primarily and continued with theoretical sampling, according to the themes as they emerged. All staff nurses who were currently working in the two major hospitals under the umbrella of the Kerman and Tehran University of Medical Sciences were considered as potential participants. The interview guide was initially developed with the help of one expert supervisor. As the study progressed, theoretical sampling was used to guide further data collection. This involved collecting more data to examine initial themes and their relationships and ensure representativeness in the categories existed by seeking additional informants for interview together with contextual data. Finally, 15 participants, comprised of female (*n* = 11) and male (*n* = 4), were interviewed during a period of 2 months. The participants were 32- 50 years of age, with a mean age of 40 years. All of them were registered nurses with a BSc degree. The mean year of service as nurses was 10 years with 7 years as mean oncology experience. All of them had experience of working with dying people and none had been educated in palliative care.

### Data collection

The main approach to collecting data was based on a qualitative method that includes narrative interviews. In-depth individual, unstructured audio-taped interviews were conducted with the participants. According to van Manen,[19] the art of a hermeneutic interview is to keep the meaning of the phenomenon open and to go on asking questions. The participants were asked to describe their experiences of being with people at the end of life. As necessary, clarifying and encouraging questions were asked such as “Can you explain a little more what you mean?” “Can you give me an example?”. The researchers, who were born in Iran, spoke in their native language (Farsi). During the interviews, the researchers attempted to strike a balance between the talk about the story by participants and keeping to the aim. The interviews were tape recorded, transcribed verbatim, and analyzed consecutively by the authors. All interviews took one session, according to participants' requests. Each session lasted between 45-55 min. Moreover, the transcripts with inferred themes were sent to the interviewees to assure that there were no ambiguities and to improve the validity of the research. All these groups of participants agreed with the themes, and in some instances they wrote additional comments that were used as data.

### Ethical considerations

The Internal Review Board of Kerman and Tehran University of Medical Sciences approved the study before data collection began. Permission, as written informed consent, was sought from the participants for the audiotape interviews and the hospital directors, Matrons, and head nurses agreed to do interviews according to a formal letter of introduction from the Vice Dean for Research of Kerman and Tehran Universities of Medical Sciences. Another ethical consideration was the assurance of confidentiality for the participants. These participants were informed about the purpose of the study and assured that their participation was voluntary. Terminally ill persons and the experiences related to them are an emotionally charged topic and may be a painful reminder of previous experiences. This risk was handled by the researchers' attentive and sensitive attitude toward the interviewees' emotional reactions. The researchers also gave the participants sufficient time to consider their participation in the study.

### Analysis

A phenomenological hermeneutic interpretation was used. From the transcribed data, a selective reading approach was adopted, implying that we read the text several times and asked “What statements or phrases seem particularly essential or revealing about the phenomenon or experience described?”. These statements were underlined and highlighted. The findings were formulated based on reflections on the essential themes that characterized the phenomenon. The hermeneutic process involved a systematic analysis of the whole text, systematic analysis of parts of the text, and comparison of the two interpretations for conflicts and an understanding of the whole compared to its parts.[19] Interpretation of the written narratives moved back and forth between the whole and parts of the texts. The goal was to find commonalities in meanings, situations, and lived experiences. Categories that clearly describe the themes were identified. The categorized themes were then translated from the native language (Farsi) into English.

### Credibility

The credibility of the findings was established by prolonged contact of more than 2 months with the participants and having other researchers independently analyze pieces of data. The coded data were then categorized and interpretations were compared. The objectivity of the data was enhanced by ensuring theoretical sensitivity whereby the researchers put aside any preconceived ideas about the topic during its analysis. The participants were also contacted after the analysis was completed to verify the interpretations of the researchers. The results were also verified with some of the staff nurses of oncology wards who did not participate in the research, and they confirmed the appropriateness or applicability of the results as well.

## RESULTS

The results identified the meaning of caring for dying people with three subthemes: (1) Being attentive to the dying persons and their families, (2) Being cared for by the dying persons and their families, and (3) Being faced with barriers. The three subthemes were comprehended as a main theme namely “sharing space and time to be lost within an organizational context” [Table 1].

**Table 1:** The overview of three subthemes and a theme

Subthemes	Theme
Being attentive to the dying persons and their families	
Being cared for by the dying people and their families	Sharing space and time to be lost within an organizational context
Being faced with barriers	

### Being attentive to the dying persons and their families

Nurses' narratives revealed that they had different ways to be attentive including relieving the spiritual pain by talking and listening, using humor and touch, and supporting dying persons and their families. The texts revealed that dying persons and their families suffered from the implication of their destiny and fluctuation of spirits (high/low spirits). Mental ambivalence among these groups of persons and their families was the most common experience that nurses confronted every working day. Consequently, the majority of the participants, according to their years of experience and experience-based teachings, expressed that they should be a good listener to the dying persons without any biased judgments in their language. The participants identified dying persons' sharing of themselves and beliefs through talking and listening to them. Additionally, smiling, being cheerful and speaking fairly to increase the happiness of them and their families were reported by the majority of the participants:

“So, we try to make contact with them and their families as we work; while I am injecting I make it seem fun or tell a joke.”

Nurses described that there is an interrelationship between the spiritual distress of persons and their families, the quality of communication, and necessary and expected support that should be provided. Factually, spiritual distress was acting as an intervening condition that was affecting the quality of support. Based on interview data and in-depth interpretations, the essence of problematic support among poor and rich dying persons and their families was different. The main intervening problem among the poor was economical problems that were exhausting them, while relinquishing a person's spouse and growing feeble in empathy was the foremost intervening condition. Therefore, such problems that can make persons' conditions worse lead nurses to support them in different ways by referring them to the social work services or even by supporting them themselves as commented:

*“They were not able to manage costs and that is why his father was so depressed. Some times the staff collected the money for them to pay for tickets and….”* (1)

To support the dying persons and their families, nurses sometimes play a counselor role for them. For instance, a female nurse described a situation in which a male dying person talked with her about his personal problems and how the nurse helped him to solve the problem:

*“I referred to his family and talked with them about his situation and finally I could persuade them to come to the ward and visit him.”* (11)

Supporting the dying person and even their families is not limited to the hospital. Some nurses talked about making an appointment with their families out of the hospital when they are off work. They believe that such friendly relations, mainly with young persons' families, help them to confront the problems that arise during this period of time:

*“Sometimes we make an appointment out of the hospital. Such friendly relations help them to overcome their problems, at least their psychological problems.”* (10)

### Being cared for by the dying people and their families

The text revealed that the majority of participants experienced a love and belonging condition or in-depth altruistic feeling among themselves and their clients within a mutually caring relationship. General inferences from interview data revealed that the majority of participants through working with dying persons experience a situation that stimulates them to be attentive to looking for meaning in their life. This has been reflected in their stories in different ways. Some of them stated that working with dying people leads them to forget their own problems. In other words, their view of life concerning what is important or not has been changed:

*“I can say that I realized what is important in life and some of the things I thought were important are not. That is why I am really satisfied.”* (5)

Some nurses stated that since working with end-of-life persons, their fear of death decreased and their acceptance of death increased. They felt that it is their different view of life that leads them to have a different view of death as well. They explained their experiences as a self-transcending, unifying force, which finds meaning in life, disease, and death:

*“Now this earthly life is so unvalued for me due to what I have seen here. I believe that death will come to me sooner or later.”* (3)

Most of the nurses reflected that working with people at the end of life requires ones to give their heart to the work and to put aside the self. They mentioned it being compassionate and account it as a specific and essential part of their relations with people at the end of life. The majority of participants stated that dealing with dying persons who are near to death with uncertain conditions made them more patient with them. It provided the participants with an inner strength that made them strong against personal difficulties:

*“As soon as I want to shout at my children, I remember my patients and I think that I should not be angry. I learnt to be patience.”* (7)

Doing the best for dying persons, both personally and professionally, was valued by all participants and supported in the contextual notes. The participants reflected the experience of enjoyment when alleviating the physical and spiritual pain because they did the best in preparing one for death. They consider it as a gift to be a witness to the dying persons and learn from them.

*“I think it is a gift to see the process of dying, because we're always leaning when we sit and support dying people….”* (5)

Participant nurses commented about their inspiring experiences, whereby they have witnessed the special situation. Two nurses explained their vitalized experiences and how they got energy from being the witness of the relations between the dying persons and their families:

*“The family begged her to enter the hospital, where they felt her pain could be better controlled. She died there, holding her daughters' hands.”* (13)

Two other nurses in their stories described the situations in which they witnessed “miracles.” They account these occasional miracles as inspiring situations and also as an important reason for their continuing to work with such people:

*“It was the last day of Farvardin [the first month in Persian calendar] and she was at home with her family. An important reason behind working here and why I stay in it is that sometimes miracles do happen.”* (12)

### Being faced with barriers

Participants described some interfering conditions in spite of their willingness to do the best for dying people. The participants felt discouraged, although they strongly desired to care for the people at the end of life; this is a consequence of the nurse shortage and heavy workload. The nurses have limited time to make an interaction with their patients, which is defined by the participants as a large barrier to provide effective care for dying persons:

*“We know that dying persons need us to be with them, but we really have no time, because there are many patients in the ward.”* (7)

Furthermore, the participants explained the lack of autonomy toward the dying persons whom they have cared for to be another key intervening condition. They emphasized that nurses, in spite of spending a lot of time with dying persons and being the most informed of their needs, are not able to intervene in the decisions making to provide the dying persons' well-being:

*“We spend a lot of time with patients but we cannot decide about their environment, about their food, and their clothes.”* (3)

The lack of a desirable environment was perceived by nurses as a barrier to their effective care for dying persons. According to them, terminally ill persons who are psychologically depressed and are hospitalized for a long time need a favorable environment with some entertainment in order to reduce their suffering and also to improve the quality of care. Without such an environment it was said:

*“Many of our patients are so depressed. They tell us that being in a hospital for a long time is so boring for them.”* (1)

The lack of palliative care to focus on people's special needs during the process of death and dying was found to be another barrier as discussed by the participants. They described that in the oncology unit, they just use chemotherapy or other life-saving strategies that lead dying persons to face an uneasy death. Palliative care, however, provides them an opportunity to help persons to overcome the fear and sadness as natural responses to death. One of them expressed her own experience with a person who was close to death, but still expected something to be done for her. It was difficult for her to accept her own death:

*“She was at the end. Her eyes were insistent with me to understand. I felt terrible that I still could not do anything for her.”* (5)

The other participants described that palliative care is providing a good quality of life with respect to the dying process and death, while using life-saving strategies in an oncology unit is actually prolonging life, but with no quality:

*“Actually we just try to extend their life with chemical drugs, even if we see how they suffer from those drugs and how many difficulties they have.”* (9)

They explored that dying persons in an oncology unit are not able to choose their treatment and have no authority to decide about the sedative drugs, while in palliative care, the persons and their families have the right to participate in every decision-making process:

*“They at least should be able to use sedative drugs, but the physician is the one who decides even about using sedative.”* (4)

It is worth noting that some nurses expressed that palliative care unit is essential for dying persons, but it is difficult for them and their families to be there and know that they have at the end. It may cause great stress to the dying persons, and may even hasten death by destroying hope. In addition, they described that while most of the dying persons would prefer to spend their dying process at home, rather than in an oncology setting, being in a palliative care unit would be a terrible experience for them. They emphasized the necessity of public education about end-of-life care in order to facilitate the people's acceptance of it:

*“But at least for some patients, and even their families, it is so hard to be in a palliative care unit. I think all the people need to be educated about end-of-life care.”* (8)

### Sharing space and time to be lost within an organizational context

According to the interview text, caring for dying people seems to mean sharing space and time to be lost within an organizational context. It is an opportunity that presents itself when the final bell in life is about to be rung. Caring for dying people was expressed by nurses as a reciprocal agreement in which they gave and received care and satisfaction through their interactions. The existential context demanded nurses to create a close relationship with dying person and the whole family, whereas the organizational context required a technical caring. It means that nurses used their efforts in forming a caring relationship in a frenzied caring environment. Nurses described the need for a special unit to focus on the caring relationship with dying persons and their families. In such an environment, they felt that they would be able to give holistic care to the patients and facilitate their dying process.

## DISCUSSION

The study revealed that all participant nurses experienced caring for dying people as an attempt to establish a caring relationship instead of technical caring. Sand and Strang[3] emphasize that respect, empathy, and provisional care with mutual togetherness and belonging may decrease the perception of existential loneliness for dying persons and their families. Several studies[2,20–23] revealed the importance of the relationships between nurses and the persons who are dying.

The nurses in this study experienced being attentive to the dying persons by talking and listening, using humor, and touch as effective approaches in order to relieve their spiritual pain. The finding is in line with previous studies in which patients identified nursing approaches for spiritual needs, including respect, talking and listening, touch and using humor.[24,25] Based on the nurses' experiences, to be attentive to the dying persons' needs also requires involvement with their family to support them emotionally and even economically. In this regard, the others also found that caring for people at the end of life extended to the family and the nurses became part of the persons' families.[21,26] Therefore, as is revealed in this study, nurses' socioeconomic interventions, together with making an emotional or friendly relationship with dying persons and their families, have been formed in order to help them to confront the problems that arise during this period of time.

Equally, participants found caring for dying persons as an opportunity that provides them personal and professional growth. This finding seems to be related with Wade's definition of self-transformation as “a dynamic, uniquely individualized process of expanding consciousness whereby an individual becomes critically aware of old and new self-views to integrate these views into a new self-definition”.[27] In other word, this form of caring relationship allows for interconnectedness, learning, growth,[28] and conceiving a new way to care for people at the end of life.[29] Moreover, nurses felt vitalized or inspired by being the witness of some special dying persons and their families' relations and also when dying persons recovered from their disease through a “miracle”. This is in line with the Mok and Chiu's[30] view that being invited into some of the most vulnerable moments of people's lives, as well as the personal meaning gained from the encounters, had enriched the nurses' life experiences. By the way, being valued by the care and interpersonal relationship led nurses to see their value as an effective nurse. Olthuis *et al*.[31] also described that the responses that nurses receive from dying persons and their families make nurses feel valued and enable them to see the value of the work they do.

On the other hand, nurses faced with some barriers while caring for persons at the end of life. For instance, being overworked due to the nurse shortage in the system and being underpaid has negative effects on their interaction with dying persons. Several studies confirmed the importance of the nurse and dying person interaction, and the time that is required to create trust and close relationships with dying persons and their families.[10,30] In addition, the nurses felt ignored at all steps of the decision-making process regarding the dying persons, even when they were the most informed about the dying persons' needs due to their frequent contact with them. This can be related to the influences of an Iranian sociocultural context on the nursing profession. According to other reports, although nursing has been perceived as a holy and honorable task among people, the culture and organizational structure being “physician-centered” still negatively affect nurses' self-confidence and authority.[11]

Furthermore, participants experienced some institutional barriers, which affected their relationship with dying persons. One of these seems to be the disregarding the dying persons' rights to have a desirable environment. This agrees with the findings in Joolaee *et al.*[32] study, where the persons experienced facility and equipment limitations in addition to inappropriate hospital structures as a barrier to fulfillment of their own rights. Another institutional barrier to the nurse and dying persons' interaction, experienced by the participants, seems to be the lack of a palliative care unit. They had different feelings about it, but all of them experienced the necessity of a patient-centered care unit in order to focus on the people's quality of life at the end rather than on the life-prolonging technologies. This echoes the other findings namely that “the opportunities to relieve suffering and help persons achieve meaningfulness from life-limiting conditions are neglected”[10] because of the dominance of technology and treatment concerns in the clinical healthcare system dealing with dying persons.[33] The finding may also reflect the philosophy among healthcare systems, which implies death as a preventable phenomenon; therefore failure to prevent death is perceived as a professional failure. Furthermore, according to the experience of some of the participants, people need to be educated and culturally prepared to accept the end-of-life care. In this regard, Cheraghi *et al.*[34] also described that the lack of development of end-of-life care could be related to the traditional cultural system in which, for instance, people prefer to die at home with family around them rather than in hospitals.

## CONCLUSION

This study suggests that the nurses' success in caring for people at the end of life depends on their interpersonal caring relationship in which they not only give the care but also drive personal growth and satisfaction. Such caring relationships as a holistic professional care can be improved by establishing palliative care to focus on end-of-life care. This establishment requires incorporation of palliative care into undergraduate nursing studies to emphasize that the process of dying is an important stage of life. It also requires cultural preparation and public education through the media, and by well-educated staff as well. The more education nurses have, the better their ability to observe their own/patients' rights and to improve quality of care, as well as ultimately quality of life at the end. These findings should be further extended by studying how dying persons and their families experience their relations with nurses in oncology units.
